# Unplanned Regionalization and Interstate Dependence in Pediatric Hospital Care

**DOI:** 10.1001/jamahealthforum.2025.6800

**Published:** 2026-02-13

**Authors:** Urbano L. França, Michael L. McManus

**Affiliations:** 1 Division of Critical Care, Department of Anesthesiology, Critical Care and Pain Medicine, Boston Children’s Hospital, Boston, Massachusetts; 2 Harvard Medical School, Boston, Massachusetts

## Abstract

**Importance:**

Pediatric hospital care is rapidly consolidating in the US, with some states now depending on neighboring states to supply pediatric inpatient services. The extent and nature of this interstate dependence have not been characterized.

**Objective:**

To describe cross-state pediatric hospital utilization patterns and quantify the degree of interstate dependence in the New England region.

**Design, Setting, and Participants:**

This was a retrospective cross-sectional study of inpatient admissions at all hospitals admitting children (age <15 years) in the New England region and the enclosing border state of New York in 2019. Admissions for mental health, routine newborn care, and pregnancy-related diagnoses were excluded. Data analysis was conducted from July to December 2024.

**Exposure:**

Hospitalization location in state or out of state.

**Main Outcomes and Measures:**

The source, number, and proportion of out-of-state admissions, bed-days, primary diagnoses, and insurance types in each state in the New England region.

**Results:**

Among 28 631 pediatric admissions, 71.1% occurred in Massachusetts, corresponding to 86.8% of bed-days in the region. Of the total admissions, 16 211 (56.7%) were of male children and slightly more than half (16 336 encounters [57.1%]) were of children older than 4 years. The fraction of residents receiving out-of-state care varied from 2.0% in Massachusetts to 65.8% in New Hampshire (median [IQR], 21.5% [11.3%-28.9%]). Massachusetts hospitals served 578 of the 599 primary diagnoses reported in the region and provided 43.5% (36 731) of all hospital days required by residents of other New England states. In all states, privately insured children were more likely than Medicaid enrollees to be admitted to out-of-state hospitals (median [IQR], 30.5% [17.0%-37.2%] vs 15.6% [6.4%-24.7%]; adjusted odds ratio, 2.21 [95% CI, 2.01-2.44]), often for common conditions.

**Conclusions and Relevance:**

In this cross-sectional study, pediatric care in New England constituted a de facto regionalized system, with substantial interstate movement of patients and heavy reliance on Massachusetts. These findings highlight an unplanned interdependence that may leave the system vulnerable to disruption. Regional planning and formal coordination among states may be necessary to ensure sustainable access to care. These findings may hold relevance for other regions.

## Key Points

**Question:**

To what extent do states depend on neighboring states for pediatric inpatient hospital care?

**Findings:**

In this cross-sectional study of 28 631 pediatric admissions in the New England region of the US, more than 70% occurred in Massachusetts, which provided nearly 87% of the bed-days in 2019. Out-of-state admissions ranged from 2.0% to 65.8% by state, and privately insured children were twice as likely as Medicaid-insured children to receive out-of-state care.

**Meaning:**

Pediatric hospital care in the New England region is highly regionalized, with substantial reliance on Massachusetts, suggesting that coordinated regional planning is essential to ensure system stability and access.

## Teaser

This cross-sectional study describes patterns in cross-state pediatric hospital utilization to quantify the degree of interstate dependence in the New England region of the US.

## Introduction

The past 2 decades have witnessed rapid consolidation of pediatric hospital services across the US.^1,2^ Driven by market forces, workforce constraints, and volume decline, hundreds of hospitals have ceased providing inpatient pediatric care for surgical procedures and even, the most common medical conditions.^3,4^ This response has led to a steep decline in the availability of inpatient pediatric care nationally, particularly in rural areas.^2,5^ The COVID-19 pandemic further accelerated the trend, with an additional 17% decline in the number of hospitals admitting children.^6^ This transformation threatens to heavily burden families seeking care.

In several pediatric domains, including trauma, neonatal intensive care, and critical care, the American Academy of Pediatrics has endorsed intentionally regionalized systems supported by formal triage protocols, capacity standards, and interfacility agreements.^7,8,9^ This planned regionalization relies on deliberate governance and coordinated resource allocation.^10^ In contrast, the regionalization presently occurring in general pediatric inpatient care is market driven, emerging from declining pediatric volume, low Medicaid reimbursement, and workforce shortages, rather than from intentional and purposeful system design.^11,12,13^ This unplanned regionalization has proceeded largely without regard to geography, population need, or capacity.^14^ Without the oversight structures that characterize established regional systems, consolidation may produce unanticipated gaps in local pediatric capacity and new dependencies on distant hospitals.

Meanwhile, health care in the US is primarily state governed, with states varying widely in their geographic size, population, resources, and economics. As a result, social programs and public services, including safety net and medical care, are uneven across the country and shaped by local factors.^15,16^ Frequently, these differences create complex dynamics ranging from cooperation to competiton.^17,18,19^

We hypothesized that the national consolidation of pediatric hospital services has been uneven across geographic areas, resulting in marked interstate variations in pediatric hospital utilization and unintended interstate dependencies. If so, multistate regional service coordination may soon be necessary to ensure access to care. To explore this hypothesis, we obtained comprehensive inpatient datasets from all 6 New England states (Connecticut, Maine, Massachusetts, New Hampshire, Rhode Island, and Vermont), as well as from the enclosing border state of New York. We then measured the volume, sources, and characteristics of all admissions, with special attention to the permeability of state lines to specific clinical conditions and/or health insurance types.

## Methods

The study was approved by the Boston Children’s Hospital Committee on Clinical Investigation. Because all data were deidentified, a waiver for informed consent was obtained. We followed the Strengthening the Reporting of Observational Studies in Epidemiology (STROBE) guidelines.

### States, Settings, and Data Sources

This was a retrospective cross-sectional study using 2019 all-encounter administrative databases from all 6 New England states and New York. To avoid related artifacts, we selected 2019 because it was the last complete year before the COVID-19 pandemic. State inpatient admission datasets were obtained from the Healthcare Cost and Utilization Project (Maine, New York, Rhode Island, and Vermont),^20^ the Massachusetts Center for Health Information and Analysis,^21^ the New Hampshire Department of Health and Human Services,^22^ and the Connecticut Department of Public Health.^23^ State population estimates were obtained from the 2019 US Census.^24^

### Study Participants

Because Massachusetts regulations require children younger than 15 years to be treated in pediatric facilities,^25^ we included all acute care hospital admissions for children younger than 15 years with residence in any of the 6 New England states and New York. Because of the lack of unique patient identifiers, the unit of analysis was hospital admission. Patients’ state of residence was determined from zip codes reported within the encounters. For New Hampshire, residence information included only the county within the state or non−New Hampshire residence. Hospital encounters were classified as in state or out of state based on hospital and patient location.

After mapping all principal *International Classification of Diseases, Tenth Revision, Clinical Modification* (*ICD-10-CM*) diagnoses into *Clinical Classification Software Refined (CCSR)*^26^ aggregator codes, we excluded the following from the analysis: all routine newborn admissions (CCSR codes PNL001, PNL002, and PNL008), other specified and unspecified perinatal conditions (CCSR code PNL013), hemolytic jaundice and perinatal jaundice (CCSR code PNL007), pregnancy-related diagnoses (CCSR code, PRG), and mental health or substance use conditions (CCSR codes MDB and SYM008). For presentation, all *ICD-10-CM* codes were also mapped to the newly revised *Pediatric Clinical Classification System *(*PECCS*).^27^ The absolute numbers of admissions were recorded and the state rates of admissions per 1000 children younger than 15 years were calculated using projected 2019 US Census data.

### Statistical Analysis

All analyses were conducted using Python, version 3.11 (Python Software Foundation), and open-source data science tools.^28^ We report descriptive and summary statistics for the patients’ demographic and encounters’ characteristics. For the number of admissions and bed-days, we report raw numbers and percentage of the total. Nonnormal continuous variables are reported as median (IQR). Comparisons between fraction of admissions and bed-days per insurance type were evaluated using the Wilcoxon signed-rank test for paired samples. Multivariable logistic regression analysis without interactions was performed to estimate the association between age, health insurance type, and state of residence with the odds of crossing state lines for care. Adjusted odds ratios (aORs) and 95% CI were calculated. A directed network was created using NetworkX (version 3.2.1) to visualize the flow of patients between states.^29^ *P* values were 2-tailed and statistical significance was defined as *P* < .05. Data analysis was conducted from July to December 2024.

## Results

### Permeability of the New England Border

In pediatric hospital care, the 6 New England states constituted a remarkably closed unit. Travel across the New York state border was infrequent and primarily limited to patients from upstate New York seeking care in a New England state and to patients from southern Connecticut seeking care in New York City. To quantify this effect, we included all pediatric admissions in and from New York in our datasets. Overall, there were 280 encounters for patients traveling from New England states to New York (<1.0% of all New England admissions) compared to 672 encounters for patients traveling from New York to New England. Of these, 455 encounters were for New York patients (67.7%) seeking care in Massachusetts and 217 encounters for New York patients (32.3%) seeking care in other New England states.

### Hospital Admissions in New England

The rate of hospital admission per 1000 children varied almost 5-fold among the 6 New England states, ranging from 3.7 in New Hampshire to 18.2 in Massachusetts (Table). In these states, there were 28 631 pediatric inpatient admissions, of which 20 362 (71.1%) were in Massachusetts. The number of pediatric admissions in the remaining states ranged from 413 (1.4%) in Vermont to 3999 (14.0%) in Connecticut. These admissions accounted for 375 036 bed-days in all of New England, with 325 391 (86.8%) occurring in Massachusetts hospitals (Table). Of the 28 631 pediatric admissions, 12 420 (43.3%) were female children and 16 211 (56.7%) were male children; slightly more than half (16 336 encounters [57.1%]) were of children older than 4 years. Almost all admissions were insured by either Medicaid (14 312 [50.0%]) or private payers (12 714 [44.4%]), but there was a small percentage of self-pay admissions (934 [3.3%]), and some with other payment types (671 [2.3%]).

**Table.** Characteristics of Hospital Admissions per State in the New England Region for Children Younger Than 15 Years

Characteristic	Admissions, No. (% of New England)					
	Connecticut	Maine	Massachusetts	New Hampshire	Rhode Island	Vermont
Hospital admissions	3999 (14.0)	1374 (4.8)	20 362 (71.1)	766 (2.7)	1717 (6.0)	413 (1.4)
Admissions per 1000 children, rate	6.7	6.7	18.2	3.7	10.2	4.4
Bed-days	25 534 (6.8)	8013 (2.1)	325 391 (86.8)	4685 (1.3)	9382 (2.5)	2031 (0.5)
Admission of out-of-state residents	43 (1.4)	52 (1.7)	2354 (79.0)	172 (5.8)	351 (11.8)	<11.0
Bed-days for out-of-state patients	232 (0.6)	395 (1.0)	36 731 (89.9)	1382 (3.4)	2078 (5.1)	17 (<0.1)
Age, y						
0-4	2236 (13.7)	727 (4.5)	11 715 (71.7)	457 (2.8)	962 (5.9)	239 (1.5)
5-9	740 (12.7)	333 (5.7)	4187 (72.1)	124 (2.1)	341 (5.9)	85 (1.5)
10-14	1023 (15.8)	314 (4.8)	4460 (68.8)	185 (2.9)	414 (6.4)	89 (1.4)
Sex						
Female	1734 (14.0)	571 (4.6)	8.876 (71.5)	321 (2.6)	743 (6.0)	175 (1.4)
Male	2265 (14.0)	803 (5.0)	11 486 (70.9)	445 (2.7)	974 (6.0)	238 (1.5)
Health insurance type						
Medicaid	2072 (14.5)	709 (5.0)	9854 (68.9)	404 (2.8)	996 (7.0)	277 (1.9)
Other	82 (12.2)	46 (6.9)	502 (74.8)	12 (1.8)	25 (3.7)	<11
Private	1770 (13.9)	573 (4.5)	9287 (73.0)	333 (2.6)	625 (4.9)	126 (1.0)
Self-pay	75 (8.0)	46 (4.9)	719 (77.0)	17 (1.8)	71 (7.6)	<11

### Out-of-State Admissions

In 2019, a total of 2979 children (10.4%) residing in a New England state traveled to a different New England state for hospital admission. Most children went to Massachusetts (2354 encounters [79.0%]), with those traveling to other states ranging from fewer than 11 admissions in Vermont to 351 admissions in Rhode Island (Table). These admissions occupied 40 836 bed-days, with 36 731 bed-days (89.9%) occurring in Massachusetts hospitals.

**Figure 1.**
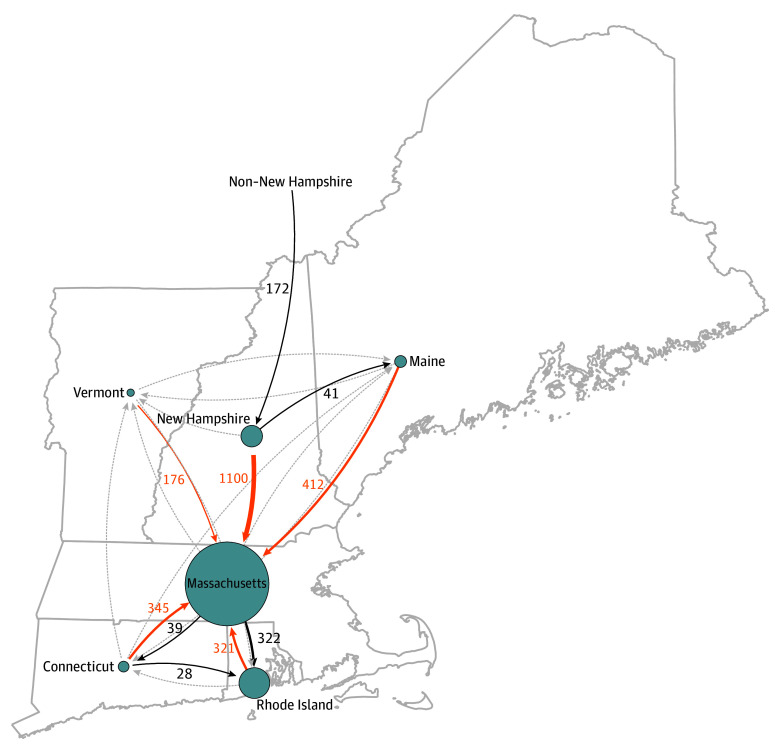
Directed Network Diagram of Out-of-State Admissions in New England in 2019 Nodes are sized by the number of out-of-state admissions served by each state. Directed edges depict the volume of admissions flowing between states. Edges with fewer than 11 encounters are not annotated. Non−New Hampshire refers to inflow of patients to New Hampshire from any other US state (see Methods and Limitations sections).

**Figure 2.**
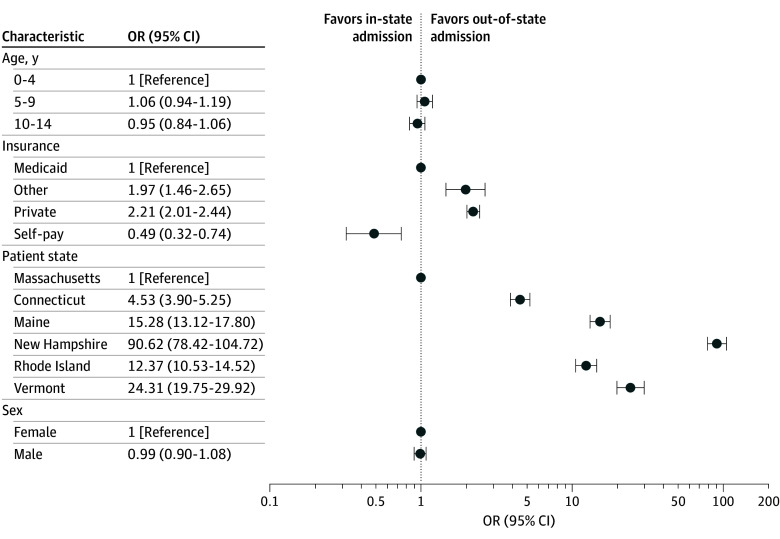
Patient Characteristics and Adjusted Odds Ratios (ORs) of Being Admitted to an Out-of-State Hospital Error bars indicate 95% CIs.

Figure 1 shows a directed network diagram summarizing the flow of patients across New England. Massachusetts served as a hub for out-of-state medical care, serving patients from all 6 states (Connecticut, 345 encounters; Maine, 412; New Hampshire, 1100; Rhode Island, 321; and Vermont, 176). Compared to Massachusetts residents, these results corresponded to aORs of being admitted outside of one’s residence state ranging from 4.53 (95% CI, 3.90-5.25) for Connecticut residents to 90.6 (95% CI, 78.4-104.7) for New Hampshire residents (Figure 2). Massachusetts residents had 322 encounters in Rhode Island, 39 in Connecticut, and fewer than 11 each in Maine and Vermont.

Among New England state residents, there was significant variability in the fraction of total hospital care provided in other New England states. This varied from 2.0% for Massachusetts to 65.8% for New Hampshire residents (state median [IQR], 21.5% [11.3%-28.9%]). Similarly, the fraction of resident bed-days provided by another state varied from 0.7% for Massachusetts to 83.0% for New Hampshire residents (state median [IQR], 44.8% [44.6%-55.8%]). Across the entire region, all hospitalizations for residents of the 5 geographically smaller New England states accounted for 84 377 bed-days, of which 36 731 (43.5%) occurred in Massachusetts.

### Clinical Diagnoses

There were 599 different *PECCS* codes listed as principal admitting diagnoses across all New England hospitals. Of these, 405 (67.6%) were listed among out-of-state admissions. The number of different admission diagnoses ranged from 578 (96.5%) in Massachusetts to 136 (22.7%) in Vermont. Of the 578 diagnoses in Massachusetts, 118 were exclusively reported by hospitals in the state. Only 83 diagnoses had admissions in all states. Diagnoses not reported in Massachusetts accounted for only 30 admissions, nearly all of which were similar to more specific diagnoses reported there (eg, cancer of brain and nervous system vs malignant neoplasm of brain or benign neoplasm of brain).

Overall, the 5 most frequent admitting diagnoses across the region were appendicitis (1426 cases), bronchiolitis (1252 cases), tonsillar hypertrophy (1081 cases), chemotherapy (930 cases), and seizures (726 cases). All but appendicitis were among the top 6 *PECCS* diagnoses for which out-of-state residents were admitted to hospitals in Massachusetts. In addition to these common diagnoses, Massachusetts hospitals most frequently admitted out-of-state New England children with congenital heart disease (321 cases), congenital musculoskeletal conditions (130 cases), brain tumors and nonseizure nervous system disorders (77 cases), congenital respiratory malformations (53 cases), extremity fractures (48 cases), cleft lip and palate (47 cases), scoliosis (39 cases), and genitourinary anomalies (34 cases). Emergent, time-sensitive conditions among out-of-state patients treated in Massachusetts included burns (34 cases), respiratory failure or arrest (30 cases), airway foreign body (14 cases), acute bowel obstruction (14 cases), and traumatic brain injury (14 cases).

### Out-of-State Admissions and Health Insurance

Of the 2979 out-of-state hospital encounters, 1065 were for Medicaid-insured encounters (35.8%), 1808 for privately insured encounters (60.7%), 30 for self-paying encounters (1.0%), and 76 for encounters with other forms of financial agreement (2.5%). These hospital admissions corresponded to 14 716 bed-days covered by Medicaid (36.0%), 24 790 by privately insured patients (60.7%), 211 by self-pay (0.5%), and 1119 by other payment type (2.7%). Privately insured patients and patients with other forms of payment were more likely (aOR, 2.21; 95% CI, 2.01-2.44; *P* < .001) than Medicaid-covered patients to cross state lines to undergo hospital admission (aOR, 1.97; 95% CI, 1.46-2.65; *P* < .001), while self-paying patients were much less likely than all groups (aOR, 0.49; 95% CI, 0.32-0.74; *P* < .001) to receive care in other states (Figure 2).

**Figure 3.**
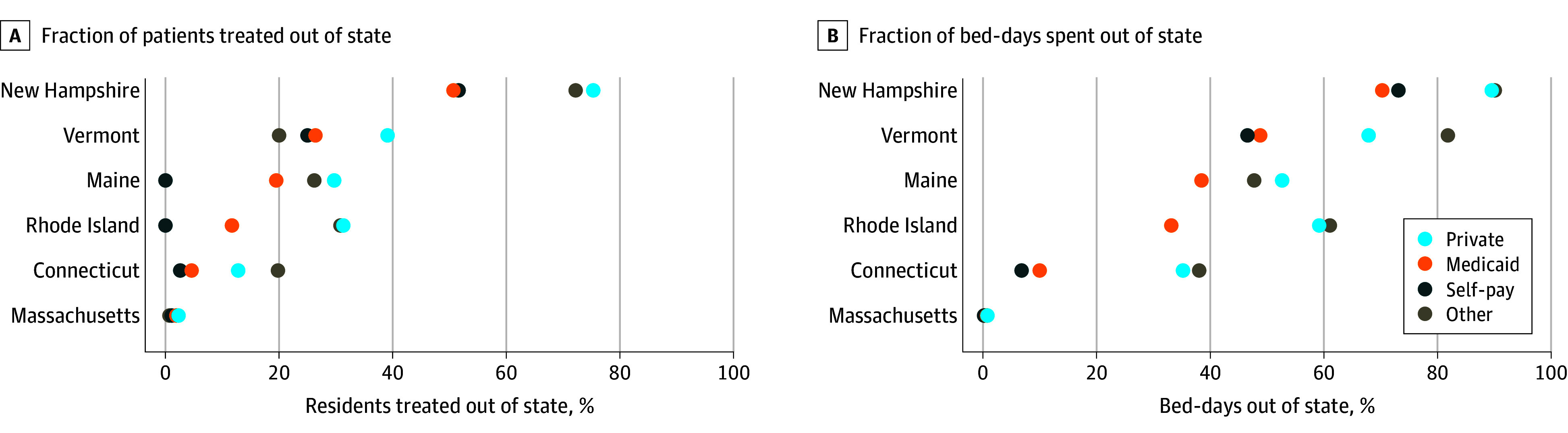
State Resident Hospital Admissions Occurring in Another State and Corresponding Bed-Days in Other States, by Health Insurance Type

Patients routinely traveled across state lines for a substantial share of their medical care, but with wide variability based on their insurance. Across all states, the median (IQR) fraction of admissions in other states by residents privately insured was almost twice that of Medicaid insured, 30.5% (17.0%-37.2%) vs 15.6% (6.4%-24.7%; *P* = .03). The same median (IQR) fraction for encounters with other forms of payment was 23.1% (19.8%-29.6%), while self-paying patients had an overall much lower median (IQR) fraction of 1.8% (0.3%-19.4%). These admissions corresponded to an even larger fraction of the total bed-days utilized by state residents. The median (IQR) fraction of out-of-state bed-days was 55.9% (39.6%-65.7%) for privately insured patients compared to 35.8% (15.8%-46.2%) for Medicaid insured patients (*P* = .03). A comparatively larger median (IQR) fraction of bed-days outside of the residing state was observed for encounters among patients with other financial agreements, 54.4% (40.5%-76.6%), and self-paying patients, 26.7% (5.1%-53.2%). Figure 3 details how these fractions varied across New England states and by health insurance type.

**Figure 4.**
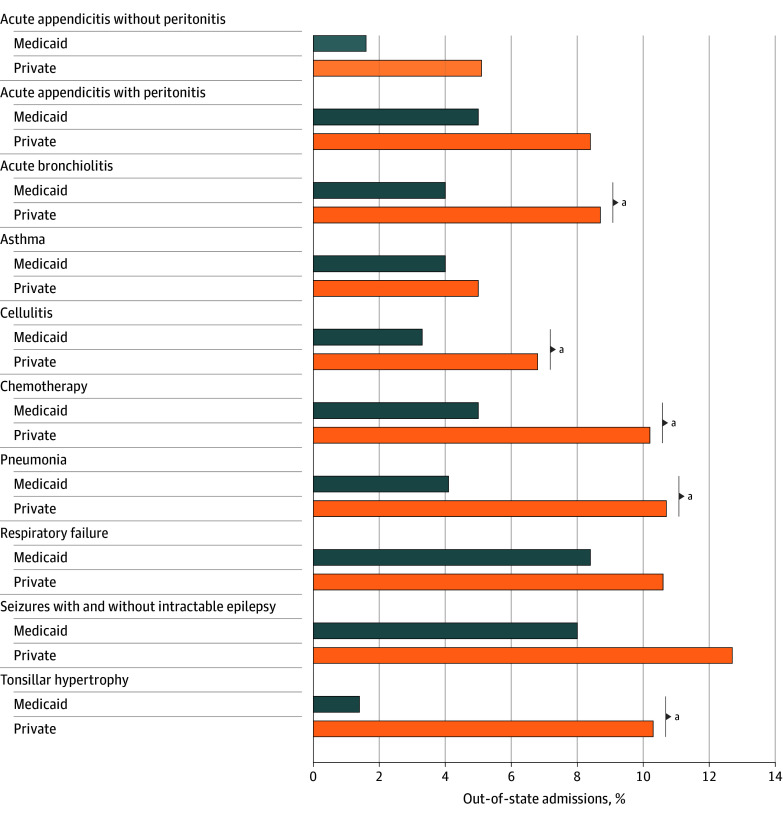
Out-of-State Admission Rates for the 10 Most Common Conditions Among Medicaid- and Privately Insured Patients in the New England Region ^a^Statistically significant (*P* < .05).

Although privately insured patients crossed state lines for care more frequently than did Medicaid patients, this frequency varied by condition. Of the conditions for which ORs reached statistical significance, privately insured patients were more likely than Medicaid-insured patients to be treated out of state for many of the most common conditions, including pneumonia, acute bronchiolitis, cellulitis, gastroesophageal reflux, benign neoplasms, dehydration, tonsillar hypertrophy, sleep apnea, vesicoureteral reflux, and failure to thrive. Figure 4 shows the Medicaid and private rates for the 10 most common conditions in all of New England. Meanwhile, Medicaid and private patients were similarly treated out of state for less common conditions, including respiratory failure, septicemia, skeletal deformities, neutropenia, and congenital heart disease.

## Discussion

With this study, we have described pediatric hospital care utilization across 6 US states. The emerging picture is one of regionalization and cooperation, with the majority of specialized services and a significant proportion of routine services provided within a single state. Across the entire region, interstate travel for care was common for both privately and publicly insured patients, with privately insured patients likely to travel more frequently and for less specialized services. The associated result is that Massachusetts, accounting for only 46.8% of the region’s children, provided 86.8% of its inpatient care days. These findings reveal an important dynamic and demonstrate that health policymakers and researchers must take a regional perspective when attempting to understand systems of pediatric care.

Both competitive and cooperative health care dynamics have been identified between and among US states. Explicit cooperation is evidenced in coordinated disaster responses, collaborative public health campaigns, and interstate licensing compacts.^30,31,32,33^ Competitive race-to-the-bottom dynamics (where states limit the scope of benefits to avoid adverse selection) have been posited in certain welfare programs^17,18^ and mutual influence is routinely evidenced in spillover dynamics, where decisions in one state (eg, Certificate of Need regulations) influence practice and behavior in its neighbors.^19,34,35^ The findings of this study reveal a local consequence of the national pediatric care regionalization dynamic: atrophy of in-state services with reliance on out-of-state services.

From a national perspective, regionalization of pediatric hospital care seems to be working well in the New England region. The current arrangement frees most states from maintaining costly, underused, and duplicative services while still maintaining access to care and high national child health rankings. For example, both the 2024 KIDS COUNT Data Book^36^ and the 2024 Health of Women and Children Report from America’s Health Rankings rank all 6 New England states among their top 16, with 3 among their top 5.^37^ The Centers for Disease Control and Prevention’s child mortality rankings^38^ are similar, with 5 states reporting among the 9 lowest rates in the nation. This success is generally attributed to strong health care systems, high rates of health insurance coverage, and robust public health policies. Notably, the New England state that is most dependent on out-of-state hospital care, New Hampshire, ranks first in the nation on composite measures of child well-being.^36,37^

While the New England region performs well on national child health metrics, aggregate outcomes may obscure growing vulnerabilities in access to care. The Andersen Behavioral Model^16^ explains that geographic, economic, and organizational barriers shape whether children can obtain needed care. New England families now travel hours to reach Massachusetts hospitals, creating geographic, financial, and logistical burdens that shape health care access for all. These burdens are most profound for those lacking transportation or confined to limited insurance networks.^39^ The high mobility of privately insured patients seeking even routine care, reflects both structural disparities and destabilizing dynamics. When privately insured patients depart and publicly insured patients remain, local facilities face adverse selection, declining pediatric volume, economic stress, and eventual loss of pediatric capability. As this capability erodes, so too does the clinical environment supporting office-based pediatric and family medicine clinicians.

Network theory teaches that systems reliant on a single node are fragile.^40,41^ In fragile systems, local disruptions can have global impacts as interruptions in key node activities trigger widespread, cascading failures.^42^ In a hospital system, disruptions may stem from countless sources, including natural disasters, financial pressures, staffing shortages, local politics, and insular management decisions. Academic medical centers, such as those dominating Massachusetts, carry additional risks associated with federal funding and trainee supply.^43,44^ Sudden cuts to Medicaid in 1 state may block access to out-of-state hospitals, and sudden nationwide cuts may force in-state hospitals to make difficult choices.^45^ Lastly, pediatric care in Massachusetts has itself been weakened by decades of intense consolidation, including the recent loss of a major children’s hospital.^46^ In systems that evolve passively, these vulnerabilities may be unrecognized until times of crisis, such as during the 2022 *triple-demic *respiratory viral season.^47,48^

If the observations of this study reflect an unintentional, market-driven atrophy and reliance dynamic, many strategies could build resilience and help maintain access across a multistate system. Data silos could be removed to facilitate the assessment of regional health services. States could coordinate Medicaid Section 1115 waivers^49^ to pool resources, share capacity, and improve cross-state continuity of care. Certificate-of-Need regulations could be aligned across state lines to prevent unnecessary duplication or erosion of services. Workforce shortages could be eased through participation in multistate licensure agreements for pediatric subspecialists. Federal grants could support regional pediatric backbone systems and infrastructure, including telehealth, emergency transport, and specialized training pipelines. Lastly, multistate compacts or US Health and Human Services−supported planning bodies could conduct joint needs assessments and resilience modeling.

### Limitations

This study has some limitations. First, it incurs all the limitations arising from administrative datasets, including potential coding errors for patient characteristics and variations in diagnostic reporting conventions. Second, we were unable to address questions regarding race and ethnicity because this information was not available in some of the datasets. Third, the Limited Use Dataset from New Hampshire does not supply zip codes for out-of-state residents. It is possible, therefore, that some out-of-state patients admitted to New Hampshire hospitals may have come from outside of the New England region. Unaccounted extraregional traffic seems unlikely, however, given that New Hampshire is bordered by other New England states, had few out-of-state admissions, and those admission were for common conditions. Fourth, because our unit of analysis was hospital encounter (rather than patient), conditions such as cancer or severe sickle cell disease could be overrepresented by single patients requiring multiple encounters. Datasets containing patients’ identifiers could be used to explore this further. Lastly, state Medicaid programs vary, so global conclusions regarding publicly insured patients may not apply to all states.

## Conclusions

In this cross-sectional study, pediatric hospital care in New England was found to function as a highly regionalized system, with all states reliant on Massachusetts for a substantial share of their inpatient services. Children with both public and private insurance routinely crossed state lines to access care, with privately insured patients more frequently doing so for common, less-specialized conditions. Given the system’s dependence on a single hub and the associated vulnerability to disruption, proactive regional planning, intergovernmental coordination, and strategic pooling of resources should be considered to ensure long-term resilience and access to care. These observations may hold relevance for other regions.

## Notes

Supplement. Data Sharing Statement

## References

[1] França UL, McManus ML. Availability of definitive hospital care for children. JAMA Pediatr. 2017;171(9):e171096-e171096. doi:10.1001/jamapediatrics.2017.1096 28692729 PMC6583506

[2] Leyenaar JK, Freyleue SD, Arakelyan M, Goodman DC, O’Malley AJ. Pediatric hospitalizations at rural and urban teaching and nonteaching hospitals in the US, 2009-2019. JAMA Netw Open. 2023;6(9):e2331807. doi:10.1001/jamanetworkopen.2023.31807 37656457 PMC10474556

[3] França UL, McManus ML. Trends in regionalization of hospital care for common pediatric conditions. Pediatrics. 2018;141(1):e20171940. doi:10.1542/peds.2017-1940 29263253

[4] McManus ML, França UL. Availability of inpatient pediatric surgery in the United States. Anesthesiology. 2021;134(6):852-861. doi:10.1097/ALN.0000000000003766 33831167

[5] Cushing AM, Bucholz EM, Chien AT, Rauch DA, Michelson KA. Availability of pediatric inpatient services in the United States. Pediatrics. 2021;148(1):e2020041723. doi:10.1542/peds.2020-041723 34127553 PMC8642812

[6] França UL, McManus ML. US pediatric inpatient care loss before and during the COVID-19 pandemic. JAMA Netw Open. 2024;7(11):e2446025. doi:10.1001/jamanetworkopen.2024.46025 39576647 PMC11584919

[7] American Academy of Pediatrics. Consensus report on the regionalization of services for critically ill or injured children. Pediatrics. 2000;105(1 Pt 1):152-155. 10617722

[8] Flynn-O’Brien KT, Srinivasan V, Fallat ME; Committee on Pediatric Emergency Medicine; Council on Injury, Violence, and Poison Prevention; Section on Critical Care; Section on Surgery; Section on Transport Medicine; Pediatric Trauma Society; Society of Trauma Nurses; Pediatric Committee. Systems-based care of the injured child: policy statement. Pediatrics. 2025;156(3):e2025072720. doi:10.1542/peds.2025-072720 40819832

[9] Stark AR, Pursley DM, Papile LA, . Standards for levels of neonatal care: II, III, and IV. Pediatrics. 2023;151(6):e2023061957. doi:10.1542/peds.2023-061957 37212022

[10] Lorch SA, Myers S, Carr B. The regionalization of pediatric health care. Pediatrics. 2010;126(6):1182-1190. doi:10.1542/peds.2010-1119 21041285 PMC3915403

[11] Colvin JD, Hall M, Berry JG, . Financial loss for inpatient care of Medicaid-insured children. JAMA Pediatr. 2016;170(11):1055-1062. doi:10.1001/jamapediatrics.2016.1639 27618284

[12] VonAchen P, Gaur D, Wickremasinghe W, . Assessment of underpayment for inpatient care at children’s hospitals. JAMA Pediatr. 2021;175(9):972-974. doi:10.1001/jamapediatrics.2021.1133 34047755 PMC8424474

[13] Krugman SD, Rauch DA. The future of inpatient community hospital care: is there one? Hosp Pediatr. 2021;11(4):422-426. doi:10.1542/hpeds.2020-003905 33727366

[14] Davis MM. The need for a national plan to sustain critical access to children’s hospital services. JAMA Netw Open. 2024;7(11):e2445972. doi:10.1001/jamanetworkopen.2024.45972 39576652

[15] Bruch SK, Meyers MK, Gornick JC. The consequences of decentralization: inequality in safety net provision in the post–welfare reform era. Soc Serv Rev. 2018;92(1):3-35. doi:10.1086/696132

[16] Andersen RM. Revisiting the behavioral model and access to medical care: does it matter? J Health Soc Behav. 1995;36(1):1-10. doi:10.2307/2137284 7738325

[17] Bailey MA, Rom MC. A wider race? Interstate competition across health and welfare programs. J Polit. 2004;66(2):326-347. doi:10.1111/j.1468-2508.2004.00154.x

[18] Choi N, Neshkova MI. Inequality and competition in state redistributive systems: evidence from welfare and health. Am Rev Public Adm. 2019;49(5):554-571. doi:10.1177/0275074018760305

[19] Volden C. States as policy laboratories: emulating success in the Children’s Health Insurance Program. Am J Pol Sci. 2006;50(2):294-312. doi:10.1111/j.1540-5907.2006.00185.x

[20] Healthcare Utilization and Cost Project. HCUP State Inpatient Databases. Accessed April 1, 2025. https://hcup-us.ahrq.gov/sidoverview.jsp

[21] Center for Health Information and Analysis. CHIA Case Mix Dataset. Accessed April 1, 2025. https://www.chiamass.gov/case-mix-data

[22] New Hampshire Department of Health and Human Services. Hospital Discharge Dataset. Accessed January 18, 2024. https://www.dhhs.nh.gov/

[23] State of Connecticut Department of Public Health. Human Investigations Committee: Hospital Discharge Dataset. Accessed June 14, 2025. https://portal.ct.gov/dph

[24] United States Census Bureau. US Census Population Data. January 18, 2024. https://www.census.gov/topics/population.html

[25] Massachusetts Department of Public Health. 105 CMR: 130.720: Requirements for all Pediatric Services (levels I-III). 2017. Accessed January 8, 2026. https://www.mass.gov/doc/105-cmr-130000-hospital-licensure/

[26] Healthcare Utilization and Cost Project. Clinical Classifications Software Refined. Accessed January 8, 2026. https://hcup-us.ahrq.gov/toolssoftware/ccsr/ccs_refined.jsp

[27] Children’s Hospital Association. Pediatric Clinical Classification System Codes. Accessed January 18, 2025. https://www.childrenshospitals.org/content/analytics/toolkit/pediatric-clinical-classification-system-peccs-codes

[28] Python Software Foundation. Python Language Reference, version 3.11. Accessed April 1, 2025. http://www.python.org

[29] NetworkX Developers. NetworkX Analysis in Python. Accessed April 1, 2025. https://networkx.org/

[30] Dixon CA. Pediatric Disaster Science: Understanding Needs, Highlighting Imperatives, and Leveraging Opportunities. National Academy of Medicine Perspectives. Accessed September 23, 2024. https://nam.edu/perspectives/pediatric-disaster-science-understanding-needs-highlighting-imperatives-and-leveraging-opportunities/10.31478/202409a PMC11784528 39896747

[31] Administration for Strategic Preparedness and Response. Regional Disaster Health Response System. Accessed April 1, 2025. https://aspr.hhs.gov/RDHRS/Pages/default.aspx

[32] Health Resources and Services Administration. Multi-state licensing compacts. Accessed April 1, 2025. https://telehealth.hhs.gov/licensure/licensure-compacts

[33] Bourdeaux M, Sasdi A, Oza S, Kerry VB. Integrating the US public health and medical care systems to improve health crisis response. Health Aff (Millwood). 2023;42(3):310-317. doi:10.1377/hlthaff.2022.01255 36877904

[34] Oyun G. Interstate spillovers, fiscal decentralization, and public spending on Medicaid home- and community-based services. Public Adm Rev. 2017;77(4):566-578. doi:10.1111/puar.12639

[35] Wang Z. The convergence of health care expenditure in the US states. Health Econ. 2009;18(1):55-70. doi:10.1002/hec.1343 18273915

[36] The Annie E. Casey Foundation. 2024 Kids Count Data Book. Accessed January 18, 2025. https://www.aecf.org/resources/2024-kids-count-data-book

[37] United Health Foundation. America’s Health Rankings: 2024 Health of Women and Children Report. 2024. Accessed May 15, 2025. https://www.americashealthrankings.org/learn/reports/2024-health-of-women-and-children-report

[38] United Health Foundation. America’s Health Rankings: Child Mortality by State. Accessed May 15, 2025. https://www.americashealthrankings.org/explore/measures/child_mortality_a

[39] Colvin JD, Hall M, Thurm C, . Hypothetical network adequacy schemes for children fail to ensure patients’ access to in-network children’s hospital. Health Aff (Millwood). 2018;37(6):873-880. doi:10.1377/hlthaff.2017.1339 29863927

[40] Elliott M, Golub B. Networks and economic fragility. Annu Rev Econ. 2022;14(1):665-696. doi:10.1146/annurev-economics-051520-021647

[41] Artime O, Grassia M, Domenico MD, . Robustness and resilience of complex networks. Nat Rev Phys. 2024;6(2):114-131. doi:10.1038/s42254-023-00676-y

[42] Valdez LD, Shekhtman L, Rocca CEL, . Cascading failures in complex networks. J Complex Netw. 2020;8(2):cnaa013. doi:10.1093/comnet/cnaa013

[43] Walensky RP, McCann NC. Challenges to the future of a robust physician workforce in the United States. N Engl J Med. 2025;392(3):286-295. doi:10.1056/NEJMsr2412784 39813651

[44] Cutler DM, Glaeser E. Cutting the NIH-The $8 Trillion Health Care Catastrophe. JAMA Health Forum. 2025;6(5):e252791. doi:10.1001/jamahealthforum.2025.2791 40440048

[45] Park E. Medicaid on the chopping block. N Engl J Med. 2025;393(11):e17. doi:10.1056/NEJMp2501855 40009824

[46] Massachusetts Health Policy Commission. Consolidation and closures in the Massachusetts pediatric health care market. 2023. Accessed December 1, 2025. https://masshpc.gov/sites/default/files/Pediatric-Policy-Report.pdf

[47] Ijaz N, Radu C, Rothenberg C, Janke AT, Venkatesh AK. Pediatric bed capacity, bed strain, and load imbalance during the 2022 respiratory viral season. JAMA Netw Open. 2025;8(9):e2533943. doi:10.1001/jamanetworkopen.2025.33943 41004148 PMC12475943

[48] Winthrop ZA, Perez JM, Staffa SJ, McManus ML, Duvall MG. Pediatric respiratory syncytial virus hospitalizations and respiratory support after the COVID-19 pandemic. JAMA Netw Open. 2024;7(6):e2416852. doi:10.1001/jamanetworkopen.2024.16852 38869896 PMC11177168

[49] Centers for Medicare & Medicaid. Section 1115 Demonstrations. Accessed January 12, 2026. https://www.medicaid.gov/medicaid/section-1115-demonstrations

