# Supplementary material for: Unplanned Regionalization and Interstate Dependence in Pediatric Hospital Care
Source: JAMA Health Forum. 2026 Feb 13;7(2):e256800. doi: 10.1001/jamahealthforum.2025.6800 (PMC12905653; doi:10.1001/jamahealthforum.2025.6800)
Supplement: Supplement. — Data Sharing Statement [file jamahealthforum-e256800-s001.pdf]

Data Sharing Statement

França. Unplanned Regionalization and Interstate Dependence in Pediatric Hospital Care. *JAMA Health Forum*. Published February 13, 2026. doi:10.1001/jamahealthforum.2025.6800

Data
Data available: No

Additional Information
Explanation for why data not available: Data Use Agreements prevent researchers from sharing the datasets used in this work. However, all of them can be obtained directly from the Healthcare and Cost Utilization Project (ME, NY, RI, and VT) and state agencies (CT, MA, NH).
